# Engineering a *Streptococcus* Cas9 Ortholog with an RxQ PAM-Binding Motif for PAM-Free Gene Control in Bacteria

**DOI:** 10.1021/acssynbio.3c00366

**Published:** 2023-08-29

**Authors:** Yuxi Teng, Jian Wang, Tian Jiang, Yusong Zou, Yajun Yan

**Affiliations:** School of Chemical, Materials and Biomedical Engineering, College of Engineering, The University of Georgia, Athens, Georgia 30602, United States

**Keywords:** CRISPR-Cas9, Cas9 engineering, PAM-free targeting, CRISPRi, gene repression

## Abstract

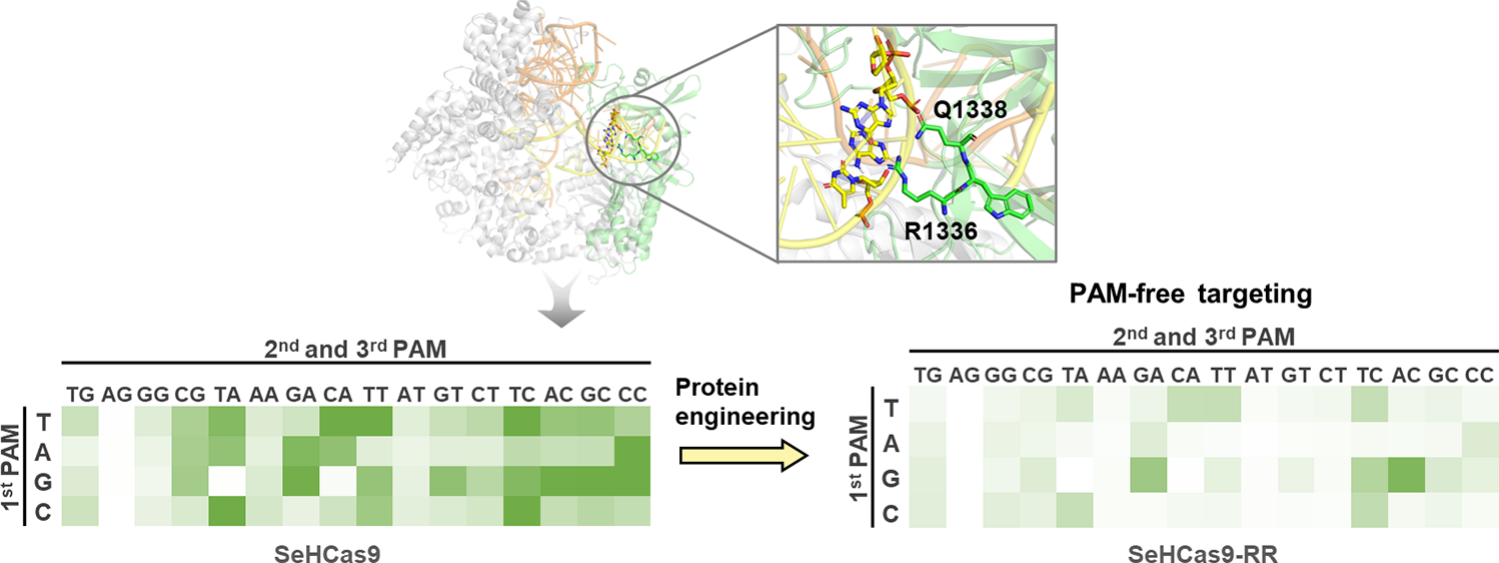

The RNA-guided Cas9 endonucleases have revolutionized gene editing and regulation, but their targeting scope is limited by the protospacer adjacent motif (PAM) requirement. The most extensively used SpCas9 from *Streptococcus pyogenes* recognizes the NGG PAM via an RxR PAM-binding motif within its PAM-interaction (PI) domain. To overcome the strict PAM requirement, we identified and characterized a Cas9 ortholog from *Streptococcus equinus* HC5 (SeHCas9) that shows high sequence identity with SpCas9 but harbors a different RxQ PAM-binding motif. Complete PAM profiling revealed that SeHCas9 recognized an NAG PAM and accommodated NKG and NAW PAMs. We investigated the PAM interaction mechanism by identifying the crucial role of R1336 within the RxQ motif in determining PAM specificity, as well as the essentiality of two conserved residues (R1152 and Q1229) across Cas9 orthologs bearing the RxQ motif for PAM recognition. Further protein engineering created two variants, SeHdCas9-Q1229R and SeHdCas9-RR, that showed robust repression across an NNG and NNN PAM range, respectively. Our work proposes a novel Cas9 PAM interaction mechanism and establishes PAM-free Cas9 variants for bacterial gene control with almost no targeting restriction.

## 1 Introduction

After the initial adaption for programmable DNA cleavage in 2012, CRISPR-Cas9 systems derived from the bacterial immune systems have sparked the rapid growth of numerous versatile CRISPR-Cas genome editing and regulation tools.^1−5^ The RNA-guided targeting of Cas effectors relies on the base pairing between the spacer sequence (∼20 nt) at the 5′ end of sgRNA and target DNA, as well as the recognition of a protospacer adjacent motif (PAM) flanking the target DNA by Cas proteins.^1,6,7^ This straightforward base pairing has endowed the CRISPR-Cas system with unparalleled programmability, with the PAM requirement being the only constraint on its targeting range. Of the diverse CRISPR-Cas systems, the dominant Class 2 Type II Cas9 with robust activities across various cell types and organisms represents the simplest PAM requirement.^1,3,4,8−12^ Nevertheless, the PAM limitation remains a significant impediment to the applicability of Cas9-based tools, especially in scenarios requiring precise targeting, such as CRISPRa, fine-tuned regulation, and base editing.^12−15^

The prototype Cas9 from *Streptococcus pyogenes* (SpCas9) strictly recognizes an NGG PAM (where N refers to A, T, G, or C). To circumvent the PAM limitation of Cas9, pioneering SpCas9 mutants VQR, EQR, VRER, and QQR1 were first generated with altered PAM specificities, respectively, toward NGA, NGAG, NGCG, and NAAG.^16,17^ Further advances in SpCas9 engineering have led to the relaxation of PAM requirement to one single-G in SpCas9-NG^12^ and xCas9-3.7^15^. The discovery of new Cas9 orthologs has also promoted Cas9 PAM expansion.^18−21^ Single-G PAM requiring variants Sc^++^^22^ and SeCas9-NQ^23^ were engineered, respectively, using the PAM-relax Cas9 orthologs ScCas9 (from *S. canis*) and SeCas9 (from *S. equinus*). Chimeric Cas9s recognizing NAA or N_4_C PAMs were created, respectively, as a result of the identification of SmacCas9 (from *S. macacae*)^24^ or SmuCas9 (from *Simonsiella muelleri*).^25^ Currently, the two most PAM-less variants, SpRY^8^ and SpdNG-LWQT,^11^ can recognize all PAMs, yet both exhibited compromised overall activities and lesser efficiency on NYN PAMs (where Y refers to T and C).^26,27^

**Figure 1:**
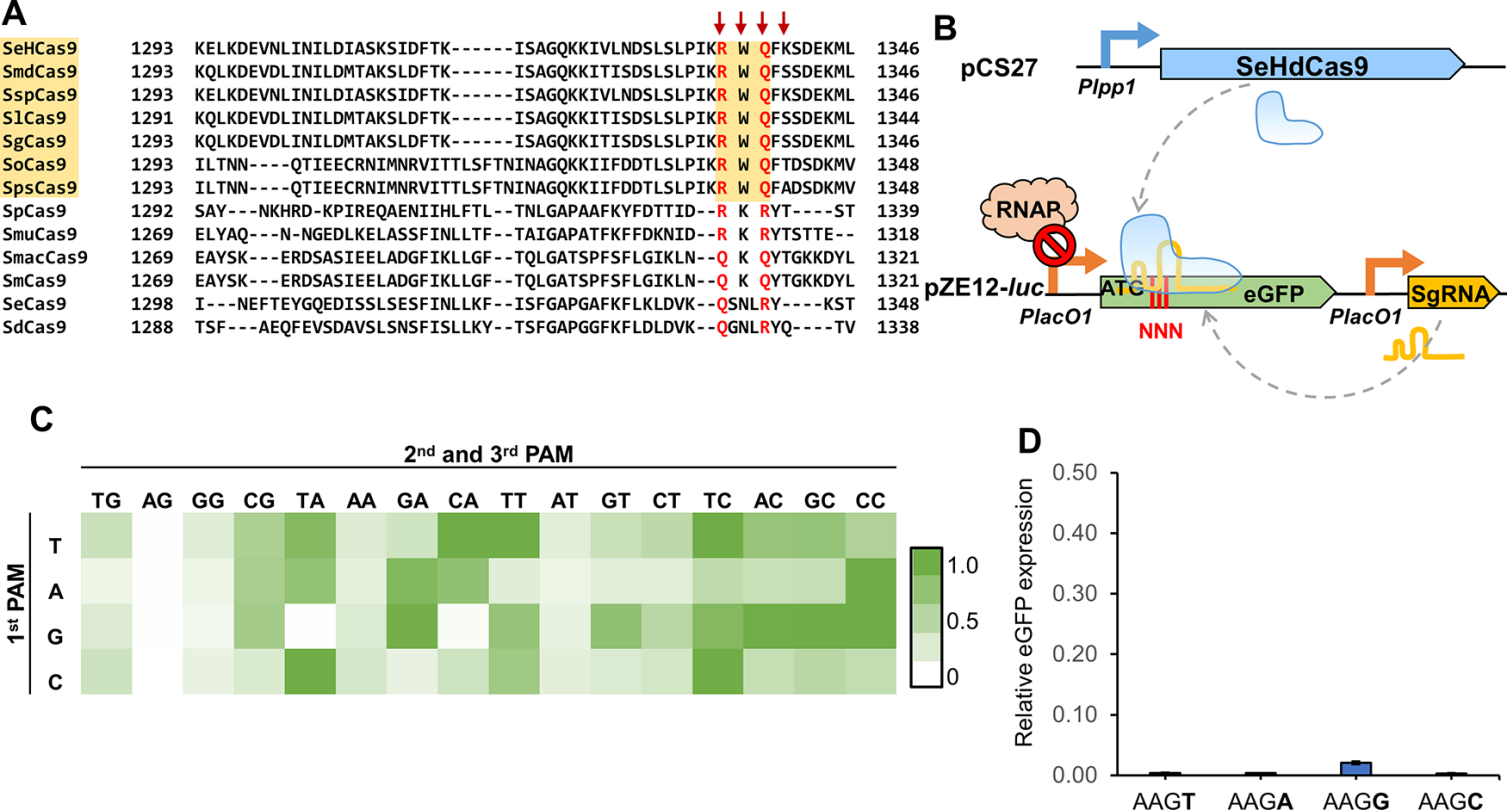
Characterization of SeHdCa9 with the RxQ PAM-binding motif. (A) Multiple sequence alignment of SpCas9-like orthologs bearing different PAM-binding motifs. (Red arrows indicate the residues selected for examination during mechanism investigation in Section 2.2). (B) The eGFP repression system: pZE12-*luc* harbors eGFP with randomized NNN inserted after the ATG start codon of eGFP and sgRNA, and pCS27 contains the catalytically deactivated SeHdCas9. The sgRNA was designed to adopt the NNN trinucleotides as a PAM. (C) Compete PAM profile of SeHdCas9 using the eGFP repression assay. (D) Impact of changing the fourth base following the NAG PAM on SeHdCas9 activity. All the *in vivo* tests were performed with three independent biological replicates.

In this study, we sought to generate robust PAM-less Cas9 variants with higher activities by exploring a Cas9 ortholog that demonstrated the potential to accommodate the most diverse PAM range among all the SpCas9-like orthologs in our previous study.^23^ This Cas9 ortholog from *S. equinus* HC5 (SeHCas9) possesses an unprecedented RxQ PAM-binding motif within the PAM-interaction (PI) domain (Figure 1A). We first evaluated the complete PAM profile of the SeHCas9 and found that it primarily favors an NAG PAM while also efficiently accepting NKG and NAW PAMs (where K refers to T or G and W refers to A or T). Mechanism dissection suggested that the NAG PAM specificity is solely determined by R1336 in the RxQ motif, and the DNA binding is additionally stabilized by two residues R1152 and Q1229, which are highly conserved in orthologs bearing the RxQ motif. Engineering SeHCas9 resulted in the generation of a PAM-free variant SeHdCas9-RR with substantially higher activities ranging from 77 to 92% on NNN PAMs and a complimentary SeHdCas9-Q1229R variant with over 96% activities on NNG PAMs. This study investigated and exploited a Cas9 ortholog with an unexplored RxQ PAM binding motif, proposing a novel PAM interaction mechanism and enabling the creation of more efficient PAM-independent Cas9 variants with broad applications in CRISPR/Cas9-based technologies.

## 2 Results

### 2.1 Profiling the PAM Preference of SeHCas9

We previously studied the diversity of PAM-binding motifs among *Streptococcus* Cas9s.^23^ Among over 500 putative *Streptococcus* Cas9s with more than 50% residue identity to SpCas9, nearly 87% of orthologs bore the RxR PAM-binding motif. The under-represented RxQ, QxQ, and QxxxR motif-bearing Cas9s accounted for only 3.4, 2.6, and 7.2%, respectively, yet can recognize different PAMs than the canonical NGG by the RxR motif. The QxQ and QxxxR motif-bearing Cas9s have been characterized to afford NAA and NAG PAM specificity and were further utilized to engineer variants with successful PAM expansion.^23,24^ However, the RxQ motif-harboring Cas9 orthologs remain largely uninvestigated but have exhibited the potential to accommodate more PAMs than the other orthologs.^23^ Further exploration identified the existence of the RxQ motif in multiple species, including the SeHCas9 from *S. equinus* HC5 (KEY47635.1) reported in our previous study,^23^ as well as SmdCas9 from *S. macedonicus* (WP_214302110.1), SspCas9 from *S. sp.* (HHU65664.1), SlCas9 from *S. lutetiensis* (WP_020917064.1), SgCas9 from *S. gallolyticus* (WP_247945562.1), SoCas9 from *S. oralis* (MBU6863003.1), and SpsCas9 from *S. pseudopneumoniae* (WP_049510439.1) (Figure 1A, Figure S1).

To completely determine the PAM profile of the RxQ motif-bearing Cas9, we employed the eGFP repression system established in our previous studies (Figure 1B).^11,23^ Specifically, we used SeHdCas9 that was constructed by swapping out the PI domain of SedCas9 with that of SeHdCas9.^23^ We also employed the Sp-sgRNA with general applicability to the SpCas9-like *Streptococcus* Cas9s^23^ and is highly similar to the SeH-sgRNA (Figure S2). A library of 64 individual pZE-NNN-eGFP-sgRNA plasmids with NNN PAM sequences was used as the reporter.^11,23^ In each reporter plasmid, the sgRNA was designed to guide SeHdCas9 to the start sequence of the eGFP ORF, with a trinucleotide (NNN) sequence inserted after the start codon as the PAM. For the PAMs potentially introducing premature stop codons (TAA, TAG, and TGA), the sgRNA was designed to adopt the corresponding PAMs closest to the starting codon of eGFP (5 bp, 44 bp, and 47 bp downstream of the start codon, respectively). Efficient PAM recognition would consequently lead to eGFP repression.

To our surprise, the repression results showed that SeHdCas9 with the RxQ PAM-binding motif recognized a broad range of PAMs, including the most favored NAG PAM, and actively recognized NKG and NAW PAMs (Figure 1C). In addition, SeHdCas9 consistently recognizes NAG PAM sequences with an efficiency of nearly 100%, regardless of the fourth PAM nucleotide (Figure 1D), which ensures that the NNN PAM library is adequate for SeHdCas9. Overall, the SeHdCsa9 exhibited a PAM recognition pattern that is more diverse than previous Cas9 orthologs.^1,18,23,24,28,29^

### 2.2 Investigating the PAM Interaction Mechanism

**Figure 2:**
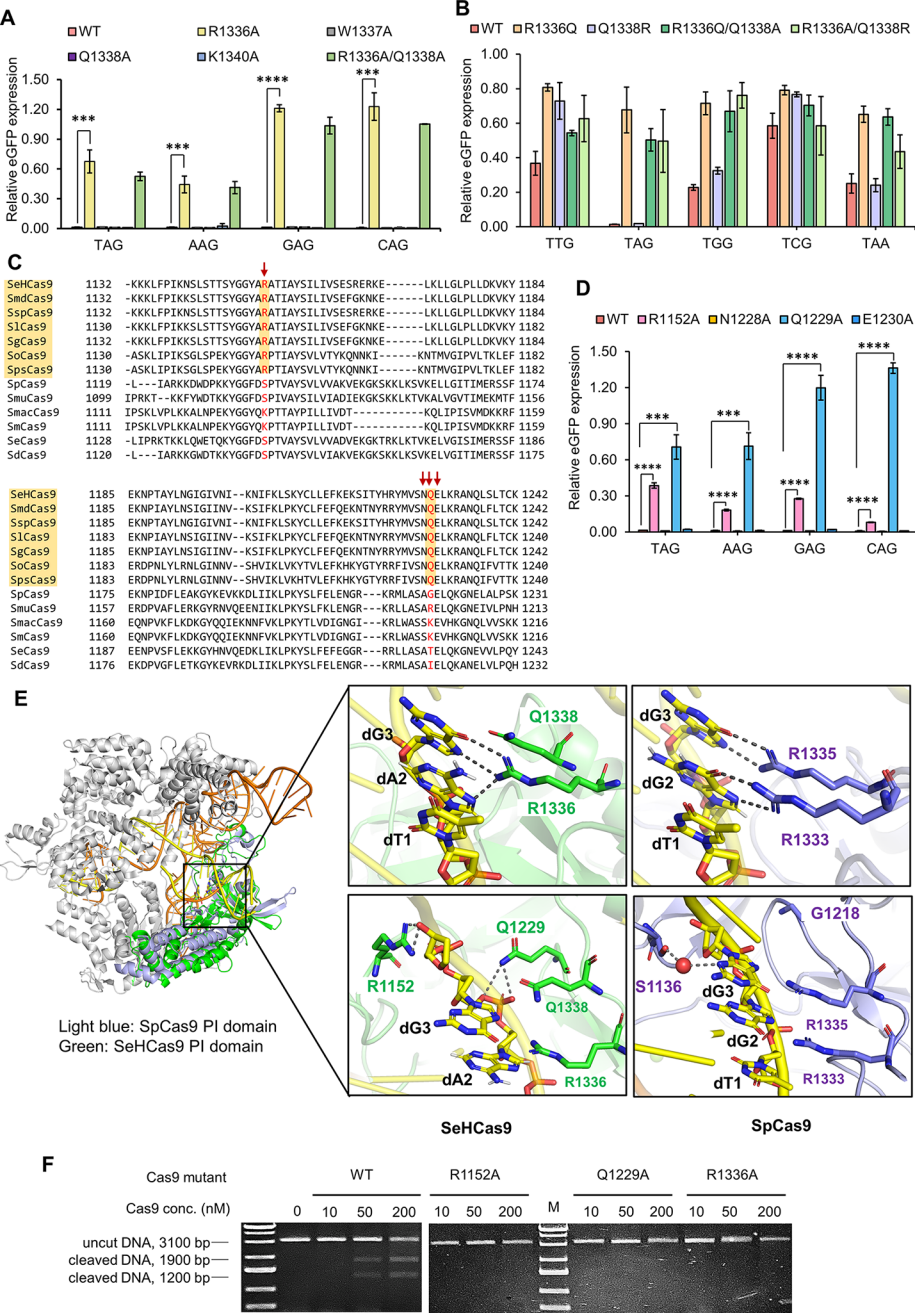
Investigation of the PAM interaction mechanism of SeHCas9. (A) Impact of residue mutagenesis within the RxQ PAM-binding motif on SeHdCas9 activity. (B) eGFP repression efficiency of R1336- and Q1338-mutants on representative NNG and NAA PAMs. (C) Sequence alignment focusing on the critical residues R1152 and Q1229 that are highly conserved among orthologs bearing the RxQ motif. (Red arrows indicate the residues selected for examination during mechanism investigation). (D) Impact of PAM-proximal residue mutagenesis on SeHdCas9 activity. (E) Overlay of the structure model of the SeHCas9 PI domain (green) with the SpCas9 PI domain (light blue) in the crystal structure of SpCas9 (PDB ID: 4UN3, gray). The dsDNA is colored in yellow, and sgRNA is in orange. Zoom-in schematics illustrate the specific interactions (dotted line in dark gray) between the RxQ PAM-binding motif and NAG PAM in SeHCas9, in comparison with the interactions between the RxR motif and NGG PAM in SpCas9 (upper panel), and the contacts (dotted line in light gray) generated by R1152 and Q1229 in SeHCas9, in comparison with the contacts by their corresponding residues, S1136 and G1218, in SpCas9 (lower panel). (F) Linearized plasmid DNA cleavage by SeHCas9 and variants with critical residue mutations. All the *in vivo* tests were performed with three independent biological replicates. ***P ≤ 0.001, ****P ≤ 0.0001 (two-tailed *t*-test).

Inspired by the specific arginine–guanine and glutamine–adenine interactions presented in many previous PAM interaction studies,^17,23,24,30,31^ our initial hypothesis was that SeHCas9 managed to position the arginine (R) and glutamine (Q) in the consensus RxQ PAM-binding motif to specifically interact with the dG3 and dA2 in the NAG PAM. However, by individually disrupting R1336, W1337, and Q1338 to alanine (A), we found that only R1336A significantly hindered the NAG PAM recognition in the repression test (Figure 2A). The Q1338A mutation had no notable impact either in the wild type or R1336A context. We also examined K1340 coming after the RxQ motif for any potential interaction with the NAG PAM,^8^ but K1340A only produced negligible differences compared to the wild type (Figure 2A). These findings suggested that only the R1336 residue in the RxQ motif is involved in specific PAM interaction. To further verify this, we investigated the possibility of altering PAM specificity by substituting the residues in the PAM-binding motif. Results showed that R1336Q substantially reduced the activity on all the tested NNG and NAA PAMs with the elimination of the preference toward NAG (Figure 2B), indicating its crucial functionality. The simultaneous introduction of Q1338A with R1336Q did not produce additional changes compared to R1336Q. Q1338R also resulted in no PAM specificity change compared to the wild type, hinting that Q1338 is insignificant in PAM interaction. In agreement with this, Q1338R failed to generate the binding to replace R1336, as demonstrated by the variant Q1338R/R1336A. Taken together, residue mutagenesis strongly implies an exclusive role of R1336 in the RxQ PAM-binding motif for PAM recognition, which was also supported by the AlphaFold-predicted structure of the SeHCas9 PI domain.^32,33^ By superimposing the predicted structure with SpCas9 (PDB ID: 4UN3), we could observe that different from the R1333 in SpCas9, the R1336 in SeHCas9 is located between dA2 and dG3 of PAMs and potentially forms hydrogen bonds with the side chains of both nucleotides (Figure 2E).

Through sequence and structural analysis, we found two critical PAM-proximal residues that are highly conserved in all orthologs with the RxQ motif, R1152 and Q1229, which correspond to S1136 and G1218 in SpCas9, respectively (Figure 2C, Figure S1). We speculated that R1152 and Q1229 possibly form extra DNA contacts for PAM recognition. By introducing R1152A and Q1229A, we observed both mutations significantly decreased the activity of SeHdCas9 on NAG PAMs, with the latter almost disrupting the recognition of NAG PAMs (Figure 2D). This result indicated that R1152 and Q1229 are crucial for PAM interaction. We also investigated the Q1229-adjacent residue N1228 that is similarly conservative in RxQ motif-bearing Cas9s, as well as the adjacent E1230 with potential contribution to the PAM interaction.^16^ However, no evidence was found to support the existence of more critical residues. Moreover, R1152A and Q1229A hindered interaction with all NNG and NAA PAMs without discrimination (Figure S3), implying that the two residues contribute to PAM recognition via non-specific interactions. These findings were also corroborated by the AlphaFold-predicted structure, in which R1152 and Q1229 may interact with the DNA backbone of PAM duplex (Figure 2E).

The importance of R1336, R1152, and Q1229 was individually validated by *in vitro* DNA cleavage (Figure 2F), which revealed that unlike the wild-type SeHCas9, the mutants with R1336A, R1152A, or Q1229A were unable to generate cleaved DNA fragments.

In summary, our investigation shows that the PAM specificity of SeHdCas9 depends solely on the R1336 residue in the RxQ PAM-binding motif, while its PAM interaction also relies on non-specific DNA interaction generated by R1152 and Q1229. This potential mechanism provides a plausible explanation for the inherent relaxation of the PAM requirement in SeHCas9, offering a distinct advantage for its engineering toward a broader PAM range.

### 2.3 Engineering SeHdCas9 for an Expanded PAM Range

**Figure 3:**
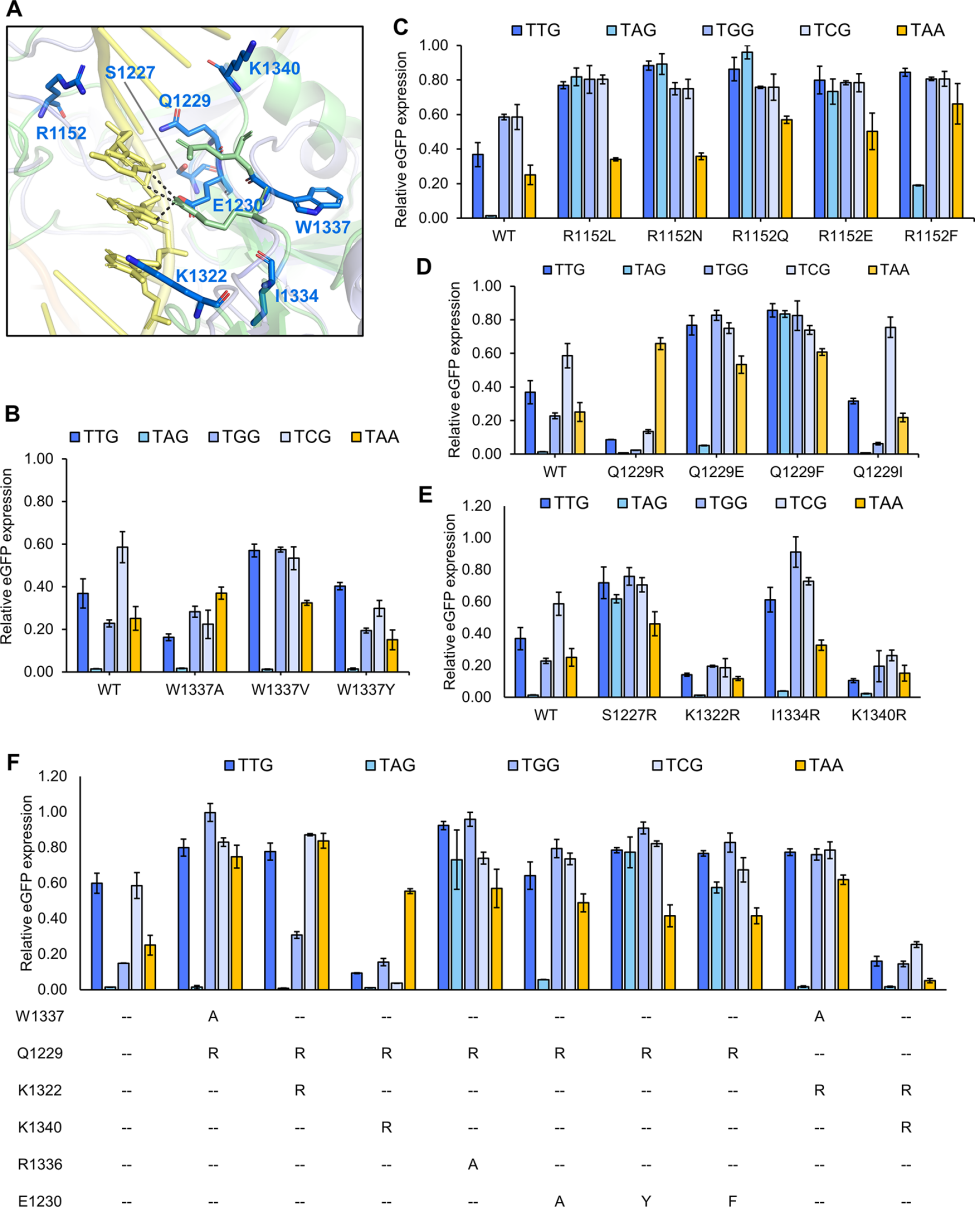
Structure-guided engineering of SeHdCas9 for PAM expansion. (A) Predicted structure of SeHCas9 with the engineering targets (colored in blue) shown. eGFP repression assay of SeHdCas9 variants with mutations on (B) W1337, (C) R1152, (D) Q1229, and (E) other residues against representative NNG and NAA PAMs. (F) Combinatorial mutations to further optimize the engineered variants. All the *in vivo* tests were performed with three independent biological replicates.

To expand the targeting scope of SeHCas9, we initially focused on the most determinant R1336 that presumably interacts with both dA2 and dG3 of the PAM. We hypothesized that enhancing its flexibility could enable recognition of a wider range of PAMs. Therefore, we mutated its adjacent bulky residue W1337 and tested the resulting variants on the NNG and NAA PAMs using the eGFP repression assay. Both W1337A and W1337Y obtained higher activities, with W1337A exhibiting over 60% repression on all the tested PAMs (Figure 3A,B).

We also explored engineering other PAM-proximal residues, starting with the two most critical auxiliary residues R1152 and Q1229. To generate stronger interactions with PAMs, we mutated R1152 to N, Q, and E with different side chains and also tested R1152L and R1152F, which potentially can create a hydrophobic environment to force PAMs toward the RxQ PAM-binding motif. However, R1152 seemed to be highly conservative, as all mutations led to substantially impaired activities on all the tested NNG and NAA PAMs, including the most preferred NAG (Figure 3A,C). We performed similar mutations on Q1229. Interestingly, Q1229R exhibited an improved repression efficiency to over 90% against almost all tested NNG PAMs but eliminated the recognition on NAA (Figure 3A,D). We also rationally engineered other residues with potential interactions with DNA, including S1227, K1322, I1334, and K1340 (Figure 3A). Surprisingly, both K1322R and K1340R led to increased recognition on all tested NNG and NAA PAMs (Figure 3E).

To optimize the engineered variants, we attempted to further expand the PAM range of SeHdCas9-Q1229R, considering its highest repression against the recognizable NNG PAMs. Specifically, we directly introduced additional beneficial mutations, including W1337A, K1322R, or K1340R, and substituted either R1336 or the R1336-stabilizing E1230^34,35^ to weaken the specific interaction between R1336 and the dG3. However, none of the mutations could further expand the NNG PAM range of Q1229R (Figure 3F), suggesting an extremely rigid PAM requirement. SeHdCas9-K1322R is another optimal variant showing the highest overall activity across all the tested NNG and NAA PAMs (Figure 3B–E). We thus combined K1322R with the advantageous W1337A or K1340R. The resulting SeHCas9-K1322R/K1340R (named SeHdCas9-RR) outperformed its parental variants with further improved overall activities on NNG and NAA PAMs (Figure 3F).

Collectively, we generated two variants, namely, SeHdCas9-Q1229R and SeHdCas9-RR, respectively, exhibiting high activity toward NNG PAM and broader PAM recognition toward NNG and NAA PAMs. The cleavage capabilities of the engineered variants were confirmed by *in vitro* DNA cleavage (Figure S4A,B). Furthermore, Q1229R, Q1338R, and K1340R exhibited high repression activities of 97 to 100% on NAG PAMs when the fourth base was randomized (Figure S5), confirming that none of them imposed any additional restriction on the fourth base of the PAM, despite their proximity to it.

### 2.4 Confirming the Complete PAM Profile of the Engineered Variants

**Figure 4:**
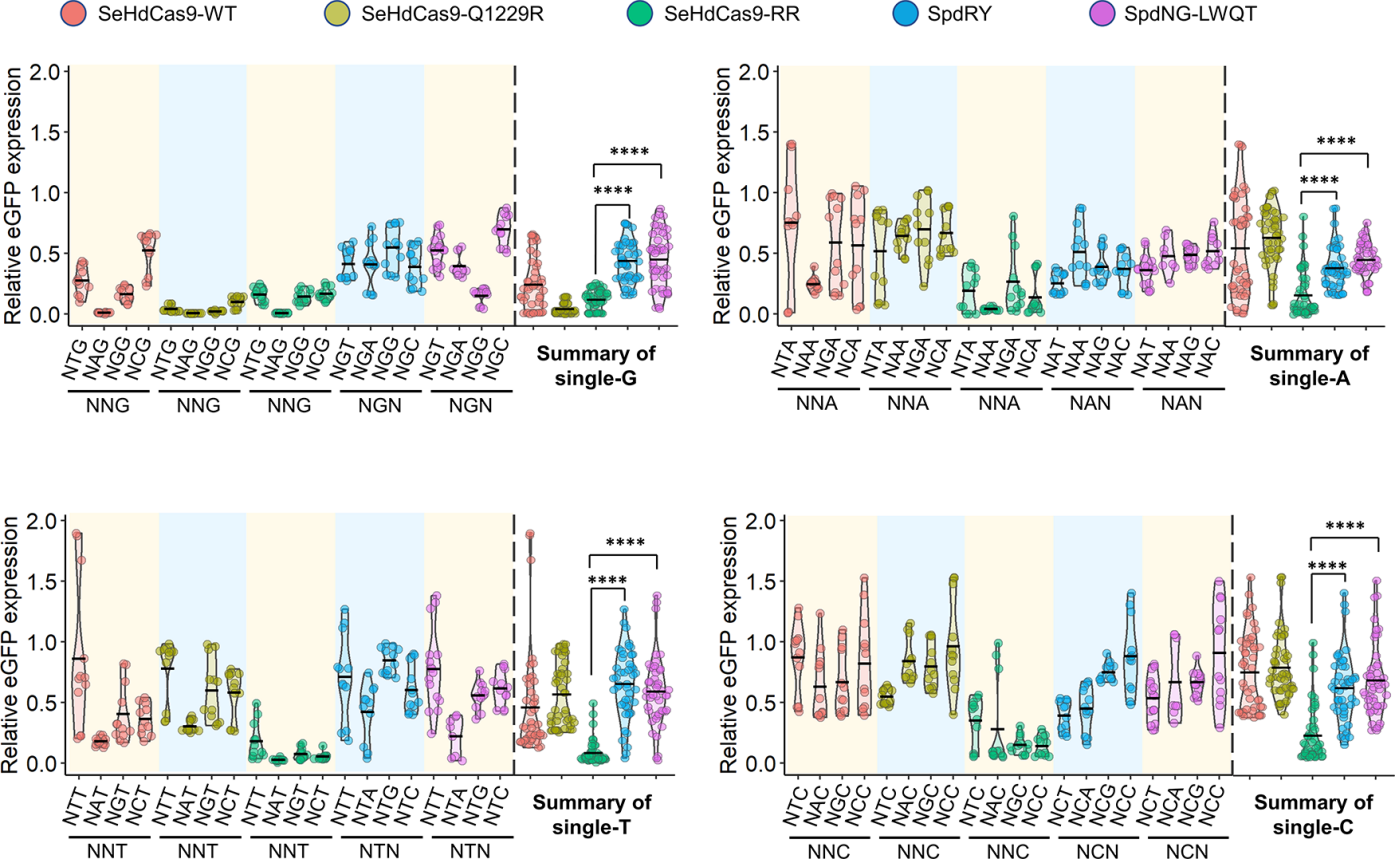
Complete PAM profile of the engineered SeHdCas9 variants, SeHdCas9-Q1229R and SeHdCas9-RR, in comparison with the SeHdCas9-WT, SpdRY, and SpdNG-LWQT on, respectively, single-G, single-A, single-T, and single-C PAMs using the eGFP repression assay. All the *in vivo* tests were performed with three independent biological replicates. Each dot represents one of the three biological replicates conducted in the repression test. ***P ≤ 0.001, ****P ≤ 0.0001 (two-tailed *t*-test).

The two engineered variants, SeHdCas9-Q1229R and SeHdCas9-RR, were examined against all NNN PAMs through the eGFP repression assay. The wild-type SeHdCas9 (SeHdCas9-WT), the most influential PAM-less SpCas9 variant SpdRY,^8^ and a PAM-flexible variant SpdNG-LWQT that we previously reported^11^ were used as controls (Figure 4).

The complete PAM profile analysis revealed that compared to SeHdCas9, both SeHdCas9-Q1229R and SeHdCas9-RR maintain a similarly high activity exceeding 99% against the canonical NAG PAMs. Meanwhile, SeHdCas9-Q1229R exhibited substantially improved recognition of all NNG PAMs with an average repression efficiency of 96% and barely any activity on other PAMs, rendering it highly specific to NNG. Regarding SeHdCas9-RR, it demonstrated a higher activity ranging from 77 to 92% on all non-canonical PAMs, with remarkable improvements on NNA, NNT, and NNC PAMs. Notably, SeHdCas9-RR outperformed SpdRY and SpdNG-LWQT on all PAMs, with over two-fold higher activity on single-T or single-C PAMs, indicating its potential to overcome the current limitations on NYN PAMs. Taken together, SeHdCas9-RR could serve as a PAM-free gene repressor with robust activity on all NNN PAMs.

## 3 Discussion

The applicability of Cas9 has been hampered by the limited targeting scope and efficiency due to the strict PAM requirement. Engineering efforts on the canonical SpCas9 and other Cas9 orthologs have largely expanded the targeting scope.^8,12,13,15^ The recent near-PAM-less SpCas9 variant SpRY achieved recognition against all NNN PAMs yet sacrificed overall robustness to some extent and exhibited less efficiency on NYN PAMs.^8^ In this study, we leveraged a promising Cas9 ortholog from *S. equinus* HC5 (SeHCas9) to develop a new Cas9 that is both PAM-less and robust. The SeHCas9 naturally adopts a highly diverse PAM range conferred by an under-investigated RxQ PAM-binding motif. The investigation into the underlying mechanism provided potential insights into the natural diversity of PAM sequences and further supported the structure-guided engineering for PAM expanding, which successfully generated a PAM-free SeHdCas9-RR that demonstrated superior repression activity on all NNN PAMs compared to SpdRY.

The proposed PAM recognition mechanism of SeHCas9 harboring the RxQ PAM-binding motif is distinct from previous Cas9 orthologs. In SpCas9 with an RxR PAM-binding motif, the two arginine residues specifically interact with the dG2 and dG3 of the NGG PAM;^17,30^ in SeCas9 with a QxxxR PAM-binding motif, the glutamine specifically recognizes the dA2 of the NAG PAM and the arginine recognizes the dG3.^23^ We initially assumed that the RxQ motif in SeHCas9 might be positioned in the opposite direction with the NAG, allowing the Q1338 to bind with the dA2 and R1336 with the dG3. However, even though R1336A eliminated the NAG PAM preference as expected, Q1338A generated no detectable influence on the preference toward dA2. Experimental and predicted results both supported the critical role of R1336 in the RxQ PAM-binding motif for PAM interaction and implied that it could simultaneously interact with dA2 and dG3 (Figure 2E). Furthermore, two PAM proximal residues in SeHCas9, namely, R1152 and Q1229, were identified, which were characterized to be essential for PAM recognition (Figure 2C,D). In SpCas9, the corresponding residues are, respectively, S1136 and G1218. S1136 forms water-mediated hydrogen bonds with dG3,^17^ and G1218 is also located proximal to the PAM duplex with the potential to form interactions by introducing mutations^16^ (Figure 2E). Our results showed that the effect of R1152A or Q1229A on PAM recognition of SeHCas9 was more pronounced compared to the corresponding mutation in SpCas9,^16^ indicating stronger contacts by the residues in SeHCas9. The contacts appeared to be non-specific (Figure S3) and likely compensate for the relatively weaker interactions by the RxQ motif. Overall, SeHCas9 with the underexplored RxQ PAM-binding motif appears to employ a novel PAM interaction mechanism, in which R1336 decides the PAM specificity, and R1152 and Q1229 generate non-specific interactions, stabilizing the PAM interaction. More precise structural information is needed in the future to fully validate the implications and elucidate this novel mechanism.

In previous efforts to engineer SpCas9, a general principle is to remove base-specific interactions and introduce non-specific protein-DNA interactions.^8,12^ Interestingly, it seems like SeHCas9 embodies this principle in its natural design, resulting in a diverse PAM range that presents a unique advantage for PAM expansion. We therefore speculated that simply introducing more non-specific interactions for SeHCas9 can further expand its PAM range. PAM-expanded SpCas9 variants have shown that mutating S1136 to tryptophan (W) with a hydrophobic side chain can push PAM toward the PAM-binding motif and strengthen their interaction.^8,11^ The corresponding residue in SeHCas9 has naturally evolved to R (R1152). Our results indicated similarly mutating R1152 to other residues with hydrophobic side chains only disrupted the activity of SeHdCas9 (Figure 3C). Moreover, any mutation on R1152 caused considerable activity loss, possibly since it is already optimal for generating the non-specific interaction required to stabilize the PAM interaction. G1218R has been extensively incorporated in many SpCas9 variants, where it can interact with the phosphate group between the third and fourth nucleotides in the PAM.^17^ Our predicted structure revealed a similar interaction generated by the equivalent Q1229 in SeHCas9, and introducing Q1229R is likely to further reinforce the interaction and improve the activity. It remains unclear why Q1229R completely altered the PAM specificity to a rigid NNG PAM. A potential explanation is that this mutation caused R1336 to strictly recognize dG3. T1337R or the equivalent mutation was also recruited in many Cas9s with an expanded PAM range,^8,11,23,24^ and could sometimes result in a preference toward G at the fourth position in PAM.^16,17^ The corresponding K1340R in SeHdCas9 also demonstrated beneficial effects on PAM expanding and has been confirmed to impose no new requirement on the fourth PAM (Figure S5). To this end, we generated a SeHCas9-Q1229R variant that is highly specific to NNG PAMs and more notably a SeHdCas9-RR variant robustly recognizing all NNN PAMs that can permit versatile gene regulations in bacteria without any restrictions. Future studies could further explore their applicability in various organisms. Moreover, the engineered variants may facilitate the development of more PAM-less genome editing tools based on additional evaluations such as *in vivo* cleavage assessment.

## 4 Methods and Materials

### 4.1 Strain and Plasmids

Supplementary Table 1 lists all the bacteria strains and plasmids in this study. *Escherichia coli* strains XL1-Blue and BL21 Star (DE3) were, respectively, used for plasmid construction and protein purification. BW25113 (F′) was the host for the eGFP repression assay. *E. coli* plasmids pCS27, pZE12-luc, and pETDuet-1 were employed for gene expression.

All DNA manipulations followed the standard molecular cloning.^36^ The restriction enzymes, Phusion high-fidelity DNA polymerase, and Quick Ligation kit were from New England Biolabs (Ipswich, MA). The plasmid pCS27-*Plpp1*-SeHdCas9 was constructed in our previous study by swapping the PI domain of SedCas9 with that of SeHdCas9.^23^ SeHdCas9-derived variants were created using a SLIM method.^37^ The NNN PAM library containing pZE-NNN-eGFP-sgRNA plasmids was developed in our previous study.^11^ SeHCas9 and its variants were cloned into pETDuet-1 plasmid between *BamHI* and *SalI* following the T7 promoter.

### 4.2 Culture Media and Conditions

*E. coli* strains were grown in Luria-Bertani (LB) medium with 5 g/L yeast extract, 10 g/L NaCl, and 10 g/L tryptone. Appropriate antibiotics (100 μg/mL ampicillin and 50 μg/mL kanamycin) and inducer isopropyl β-d-1-thiogalactopyranoside (IPTG, 0.5 mM) were supplemented as required. Cultures were incubated at 37 °C with a rotating speed of 270 rpm.

### 4.3 eGFP Repression Assay

The PAM recognition was determined as previously described.^11,23^ Briefly, individual reporter plasmid (pZE-NNN-eGFP-sgRNA) from the NNN PAM library was co-transformed with pCS27 harboring SeHdCas9 or its variants into *E. coli* BW25113 (F′). The control group consisted of each reporter plasmid with empty pCS27. Three randomly picked single transformants were inoculated to 3 mL of LB medium with proper antibiotics and IPTG inducer. After 24 h of incubation, 20 μL of culture was diluted using 180 μL of water in a black 96-well plate. A Synergy microplate reader (BioTek, Winooski, VT) was used to measure the cell density at 600 nm (OD600), as well as the eGFP fluorescence with an excitation filter of 485/20 nm and an emission filter of 528/20 nm. The relative eGFP expression was determined as the ratio of normalized eGFP fluorescence (eGFP/OD600) of the SeHdCas9-testing samples to that of the control group without dCas9. All tests were performed in biological triplicate.

### 4.4 Protein Purification

To obtain the Cas9 proteins, pETDuet plasmids containing SeHCas9 or its variants were transformed into *E. coli* BL21 Star (DE3). Single transformants were inoculated in 3 mL of LB medium and incubated at 37 °C. One percent of the overnight culture was transferred to 50 mL of LB medium in a 250 mL shake flask and grown at 37 °C. When the OD600 reached 0.6–0.8, protein expression was induced by adding 0.5 mM IPTG and the flask was incubated at 30 °C, 270 rpm for another 9–12 h. The harvested cells were subjected to disruption by a Mini Bead Beater (Biospec). Proteins were purified using a His-Spin Protein Miniprep Kit (Zymo Research) as per the manufacturer’s instructions. The obtained proteins were validated by Tricine-SDS-polyacrylamide gel electrophoresis (Tricine-SDS-PAGE) on a 12% protein gel, and the protein concentrations were quantified using a Pierce BCA Protein Assay Kit (Thermo Scientific) following the manufacturer’s instructions.

### 4.5 *In Vitro* Cleavage

The sgRNA designed for the eGFP repression assay (Figure 1B) was produced with the T7 RNA polymerase (NEB), and the product was purified using a Monarch RNA Cleanup Kit (NEB) according to the manufacturers’ instructions. The pZE-NNN-eGFP-sgRNA reporter plasmids with representative PAMs (ATG, AAG, AGG, ACG, and AAA) were linearized using *SacI* and then used as the DNA substrate. For *in vitro* cleavage, a 20 μL reaction mixture was prepared by mixing 400 nM sgRNA, 3 nM linearized reporter plasmids (120 ng) as the substrate, and varying concentrations of Cas9s (0, 10, 50, or 200 nM) in a reaction buffer containing 20 mM Tris–HCl (pH = 7.5), 100 mM NaCl, 2 mM MgCl_2_, 1 mM DTT, and 5% glycerol. The reaction was incubated at 37 °C for 30 min and then stopped by heating at 75 °C for 10 min. Cleavage results were resolved on a 1% agarose gel.

## Supporting Information Available

Strains and plasmids used in this work; sequence alignment of representative Cas9 orthologs; sequence comparison between Sp-sgRNA and SeH-sgRNA; activities of SeHdCas9-R1152A and SeHdCas9-Q1229A on representative NNG and NAA PAMs; *in vitro* cleavage results of SeHCas9-Q1229R and SeHCas9-RR against NNG and NAA PAMs; activities of SeHdCas9-Q1229R, SeHdCas9-Q1338R, and SeHdCas9-K1340R on AAGN PAMs (PDF)

## References

[ref1] Jinek M.; Chylinski K.; Fonfara I.; Hauer M.; Doudna J. A.; Charpentier E. A programmable dual-RNA-guided DNA endonuclease in adaptive bacterial immunity. Science 2012, 337, 816–821. 10.1126/science.1225829. 22745249 PMC6286148

[ref2] Anzalone A. V.; Koblan L. W.; Liu D. R. Genome editing with CRISPR-Cas nucleases, base editors, transposases and prime editors. Nat. Biotechnol. 2020, 38, 824–844. 10.1038/s41587-020-0561-9. 32572269

[ref3] Cong L.; Ran F. A.; Cox D.; Lin S.; Barretto R.; Habib N.; Hsu P. D.; Wu X.; Jiang W.; Marraffini L. A.; Zhang F. Multiplex Genome Engineering Using CRISPR/Cas Systems. Science 2013, 339, 819–823. 10.1126/science.1231143. 23287718 PMC3795411

[ref4] Jiang W.; Bikard D.; Cox D.; Zhang F.; Marraffini L. A. RNA-guided editing of bacterial genomes using CRISPR-Cas systems. Nat. Biotechnol. 2013, 31, 233–239. 10.1038/nbt.2508. 23360965 PMC3748948

[ref5] Teng Y.; Zhang J.; Jiang T.; Zou Y.; Gong X.; Yan Y. Biosensor-enabled pathway optimization in metabolic engineering. Curr. Opin. Biotechnol. 2022, 75, 102696 10.1016/j.copbio.2022.102696. 35158314 PMC9177593

[ref6] Jiang F.; Doudna J. A. CRISPR-Cas9 Structures and Mechanisms. Annu. Rev. Biophys. 2017, 46, 505–529. 10.1146/annurev-biophys-062215-010822. 28375731

[ref7] Gasiunas G.; Barrangou R.; Horvath P.; Siksnys V. Cas9–crRNA ribonucleoprotein complex mediates specific DNA cleavage for adaptive immunity in bacteria. Proc. Natl Acad. Sci. 2012, 109, E2579–E2586. 10.1073/pnas.1208507109. 22949671 PMC3465414

[ref8] Walton R. T.; Christie K. A.; Whittaker M. N.; Kleinstiver B. P. Unconstrained genome targeting with near-PAMless engineered CRISPR-Cas9 variants. Science 2020, 368, 290–296. 10.1126/science.aba8853. 32217751 PMC7297043

[ref9] Collias D.; Leenay R. T.; Slotkowski R. A.; Zuo Z.; Collins S. P.; McGirr B. A.; Liu J.; Beisel C. L. A positive, growth-based PAM screen identifies noncanonical motifs recognized by the S. pyogenes Cas9. Sci. Adv. 2020, 6, eabb4054 10.1126/sciadv.abb4054. 32832642 PMC7439565

[ref10] Knott G. J.; Doudna J. A. CRISPR-Cas guides the future of genetic engineering. Science 2018, 361, 866–869. 10.1126/science.aat5011. 30166482 PMC6455913

[ref11] Wang J.; Teng Y.; Zhang R.; Wu Y.; Lou L.; Zou Y.; Li M.; Xie Z. R.; Yan Y. Engineering a PAM-flexible SpdCas9 variant as a universal gene repressor. Nat. Commun. 2021, 12, 6916 10.1038/s41467-021-27290-9. 34824292 PMC8617050

[ref12] Nishimasu H.; Shi X.; Ishiguro S.; Gao L.; Hirano S.; Okazaki S.; Noda T.; Abudayyeh O. O.; Gootenberg J. S.; Mori H.; Oura S.; Holmes B.; Tanaka M.; Seki M.; Hirano H.; Aburatani H.; Ishitani R.; Ikawa M.; Yachie N.; Zhang F.; Nureki O. Engineered CRISPR-Cas9 nuclease with expanded targeting space. Science 2018, 361, 1259–1262. 10.1126/science.aas9129. 30166441 PMC6368452

[ref13] Collias D.; Beisel C. L. CRISPR technologies and the search for the PAM-free nuclease. Nat. Commun. 2021, 12, 555 10.1038/s41467-020-20633-y. 33483498 PMC7822910

[ref14] Huang T. P.; Zhao K. T.; Miller S. M.; Gaudelli N. M.; Oakes B. L.; Fellmann C.; Savage D. F.; Liu D. R. Circularly permuted and PAM-modified Cas9 variants broaden the targeting scope of base editors. Nat. Biotechnol. 2019, 37, 626–631. 10.1038/s41587-019-0134-y. 31110355 PMC6551276

[ref15] Hu J. H.; Miller S. M.; Geurts M. H.; Tang W.; Chen L.; Sun N.; Zeina C. M.; Gao X.; Rees H. A.; Lin Z.; Liu D. R. Evolved Cas9 variants with broad PAM compatibility and high DNA specificity. Nature 2018, 556, 57–63. 10.1038/nature26155. 29512652 PMC5951633

[ref16] Kleinstiver B. P.; Prew M. S.; Tsai S. Q.; Topkar V. V.; Nguyen N. T.; Zheng Z.; Gonzales A. P.; Li Z.; Peterson R. T.; Yeh J. R.; Aryee M. J.; Joung J. K. Engineered CRISPR-Cas9 nucleases with altered PAM specificities. Nature 2015, 523, 481–485. 10.1038/nature14592. 26098369 PMC4540238

[ref17] Anders C.; Bargsten K.; Jinek M. Structural Plasticity of PAM Recognition by Engineered Variants of the RNA-Guided Endonuclease Cas9. Mol. Cell 2016, 61, 895–902. 10.1016/j.molcel.2016.02.020. 26990992 PMC5065715

[ref18] Gasiunas G.; Young J. K.; Karvelis T.; Kazlauskas D.; Urbaitis T.; Jasnauskaite M.; Grusyte M. M.; Paulraj S.; Wang P. H.; Hou Z.; Dooley S. K.; Cigan M.; Alarcon C.; Chilcoat N. D.; Bigelyte G.; Curcuru J. L.; Mabuchi M.; Sun Z.; Fuchs R. T.; Schildkraut E.; Weigele P. R.; Jack W. E.; Robb G. B.; Venclovas Č.; Siksnys V. A catalogue of biochemically diverse CRISPR-Cas9 orthologs. Nat. Commun. 2020, 11, 5512 10.1038/s41467-020-19344-1. 33139742 PMC7606464

[ref19] Kleinstiver B. P.; Prew M. S.; Tsai S. Q.; Nguyen N. T.; Topkar V. V.; Zheng Z.; Joung J. K. Broadening the targeting range of Staphylococcus aureus CRISPR-Cas9 by modifying PAM recognition. Nat. Biotechnol. 2015, 33, 1293–1298. 10.1038/nbt.3404. 26524662 PMC4689141

[ref20] Ran F. A.; Cong L.; Yan W. X.; Scott D. A.; Gootenberg J. S.; Kriz A. J.; Zetsche B.; Shalem O.; Wu X.; Makarova K. S.; Koonin E. V.; Sharp P. A.; Zhang F. In vivo genome editing using Staphylococcus aureus Cas9. Nature 2015, 520, 186–191. 10.1038/nature14299. 25830891 PMC4393360

[ref21] Ma D.; Xu Z.; Zhang Z.; Chen X.; Zeng X.; Zhang Y.; Deng T.; Ren M.; Sun Z.; Jiang R.; Xie Z. Engineer chimeric Cas9 to expand PAM recognition based on evolutionary information. Nat. Commun. 2019, 10, 560 10.1038/s41467-019-08395-8. 30718489 PMC6361995

[ref22] Chatterjee P.; Jakimo N.; Lee J.; Amrani N.; Rodríguez T.; Koseki S. R. T.; Tysinger E.; Qing R.; Hao S.; Sontheimer E. J.; Jacobson J. An engineered ScCas9 with broad PAM range and high specificity and activity. Nat. Biotechnol. 2020, 38, 1154–1158. 10.1038/s41587-020-0517-0. 32393822

[ref23] Wang J.; Teng Y.; Gong X.; Zhang J.; Wu Y.; Lou L.; Li M.; Xie Z.-R.; Yan Y. Exploring and engineering PAM-diverse Streptococci Cas9 for PAM-directed bifunctional and titratable gene control in bacteria. Metab. Eng. 2023, 75, 68–77. 10.1016/j.ymben.2022.10.005. 36404524 PMC10947553

[ref24] Chatterjee P.; Lee J.; Nip L.; Koseki S. R. T.; Tysinger E.; Sontheimer E. J.; Jacobson J. M.; Jakimo N. A Cas9 with PAM recognition for adenine dinucleotides. Nat. Commun. 2020, 11, 2474 10.1038/s41467-020-16117-8. 32424114 PMC7235249

[ref25] Wei J.; Hou L.; Liu J.; Wang Z.; Gao S.; Qi T.; Gao S.; Sun S.; Wang Y. Closely related type II-C Cas9 orthologs recognize diverse PAMs. eLife 2022, 11, e77825 10.7554/eLife.77825. 35959889 PMC9433092

[ref26] Kiattisewee C.; Karanjia A. V.; Legut M.; Daniloski Z.; Koplik S. E.; Nelson J.; Kleinstiver B. P.; Sanjana N. E.; Carothers J. M.; Zalatan J. G. Expanding the Scope of Bacterial CRISPR Activation with PAM-Flexible dCas9 Variants. ACS Synth. Biol. 2022, 11, 4103–4112. 10.1021/acssynbio.2c00405. 36378874 PMC10516241

[ref27] Ren Q.; Sretenovic S.; Liu S.; Tang X.; Huang L.; He Y.; Liu L.; Guo Y.; Zhong Z.; Liu G.; Cheng Y.; Zheng X.; Pan C.; Yin D.; Zhang Y.; Li W.; Qi L.; Li C.; Qi Y.; Zhang Y. PAM-less plant genome editing using a CRISPR-SpRY toolbox. Nat. Plants 2021, 7, 25–33. 10.1038/s41477-020-00827-4. 33398158

[ref28] Wang S.; Tao C.; Mao H.; Hou L.; Wang Y.; Qi T.; Yang Y.; Ong S. G.; Hu S.; Chai R.; Wang Y. Identification of SaCas9 orthologs containing a conserved serine residue that determines simple NNGG PAM recognition. PLoS Biol. 2022, 20, e3001897 10.1371/journal.pbio.3001897. 36449487 PMC9710800

[ref29] Chatterjee P.; Jakimo N.; Jacobson J. M. Minimal PAM specificity of a highly similar SpCas9 ortholog. Sci. Adv. 2018, 4, eaau0766 10.1126/sciadv.aau0766. 30397647 PMC6200363

[ref30] Anders C.; Niewoehner O.; Duerst A.; Jinek M. Structural basis of PAM-dependent target DNA recognition by the Cas9 endonuclease. Nature 2014, 513, 569–573. 10.1038/nature13579. 25079318 PMC4176945

[ref31] Hirano H.; Gootenberg J. S.; Horii T.; Abudayyeh O. O.; Kimura M.; Hsu P. D.; Nakane T.; Ishitani R.; Hatada I.; Zhang F.; Nishimasu H.; Nureki O. Structure and Engineering of Francisella novicida Cas9. Cell 2016, 164, 950–961. 10.1016/j.cell.2016.01.039. 26875867 PMC4899972

[ref32] Mirdita M.; Schütze K.; Moriwaki Y.; Heo L.; Ovchinnikov S.; Steinegger M. ColabFold: making protein folding accessible to all. Nat. Methods 2022, 19, 679–682. 10.1038/s41592-022-01488-1. 35637307 PMC9184281

[ref33] Jumper J.; Evans R.; Pritzel A.; Green T.; Figurnov M.; Ronneberger O.; Tunyasuvunakool K.; Bates R.; Žídek A.; Potapenko A.; Bridgland A.; Meyer C.; Kohl S. A. A.; Ballard A. J.; Cowie A.; Romera-Paredes B.; Nikolov S.; Jain R.; Adler J.; Back T.; Petersen S.; Reiman D.; Clancy E.; Zielinski M.; Steinegger M.; Pacholska M.; Berghammer T.; Bodenstein S.; Silver D.; Vinyals O.; Senior A. W.; Kavukcuoglu K.; Kohli P.; Hassabis D. Highly accurate protein structure prediction with AlphaFold. Nature 2021, 596, 583–589. 10.1038/s41586-021-03819-2. 34265844 PMC8371605

[ref34] Kang M.; Zuo Z.; Yin Z.; Gu J. Molecular Mechanism of D1135E-Induced Discriminated CRISPR-Cas9 PAM Recognition. J. Chem. Inf. Model. 2022, 62, 3057–3066. 10.1021/acs.jcim.1c01562. 35666156

[ref35] Guo M.; Ren K.; Zhu Y.; Tang Z.; Wang Y.; Zhang B.; Huang Z. Structural insights into a high fidelity variant of SpCas9. Cell Res 2019, 29, 183–192. 10.1038/s41422-018-0131-6. 30664728 PMC6460432

[ref36] Sambrook J.; Fritsch E. F.; Maniatis T., Molecular cloning: a laboratory manual. Cold spring harbor laboratory press: 1989.

[ref37] Chiu J.; March P. E.; Lee R.; Tillett D. Site-directed, Ligase-Independent Mutagenesis (SLIM): a single-tube methodology approaching 100% efficiency in 4 h. Nucleic Acids Res. 2004, 32, e174 10.1093/nar/gnh172. 15585660 PMC535700

