# Supplementary material for: Engineering a Streptococcus Cas9 Ortholog with an RxQ PAM-Binding Motif for PAM-Free Gene Control in Bacteria
Source: ACS Synth Biol. 2023 Aug 29;12(9):2764–72. doi: 10.1021/acssynbio.3c00366 (PMC10510713; doi:10.1021/acssynbio.3c00366)
Supplement: Supplementary file 1 — sb3c00366_si_001.pdf [file sb3c00366_si_001.pdf]

Supporting Information

Yuxi Teng[a], Jian Wang[a], Tian Jiang[a], Yusong Zou[a], Yajun Yan[a, 1]

[a] School of Chemical, Materials and Biomedical Engineering, College of Engineering, The University of Georgia, Athens, GA 30602, USA.

[1] Corresponding author: Yajun Yan

Address: 2040E Interdisciplinary STEM Research Building 1, 302 East Campus Road, Athens, GA 30602, USA

E-mail: yajunyan@uga.edu; telephone: +1-706-542-8293

Supplementary Table 1. Strains and plasmids used in this study.

Strains	Description	Reference
E. coli XL-1 Blue	*recA*1 *endA*1 *gyrA*96 *thi*-1 *hsdR*17 *supE*44 *relA*1 *lac F'* [*traD36 proAB* $lacI^{q}Z\Delta M15$ *Tn*10 (Tet^{r})]	Stratagene
E. coli BW25113 (F')	*rrnBT*14 Δ*lacZWJ*16 *hsdR*514 *ΔaraBADAH*33 *ΔrhaBADLD*78 *F'* [*traD36 proAB* $lacI^{q}Z\Delta M15$ *Tn10*(Tet^{r})]	[1]
E. coli BL21 Star (DE3)	F^{-} *ompT hsdSB (rB-mB-) gal dcm rne131* (DE3)	Invitrogen
**Plasmids**	**Description**	**Reference**
pCS27	P_{L}*lacO1*, P15A ori, Kan^{R}	[2]
pZE12-luc	P_{L}*lacO1*, colE ori, Amp^{R}	[3]
pETDuet-1	T7, pBR322 ori, Amp^{R}	Novagen
pCS27-SeHdCas9	pCS27-SedCas9 with PI domain (1109-1375aa) swapped by SeHCas9 PI domain (1109-1375 aa)	[4]
pCS27-SeHdCas9-R1336A	pCS27-SeHdCas9 harboring with R1336A	This study
pCS27-SeHdCas9-W1337A	pCS27-SeHdCas9 harboring with W1337A	This study

pCS27-SeHdCas9-Q1338A	pCS27-SeHdCas9 harboring Q1338A	This study
pCS27-SeHdCas9-R1336A/Q1338A	pCS27-SeHdCas9 harboring R1336A and Q1338A	This study
pCS27-SeHdCas9-R1336Q	pCS27-SeHdCas9 harboring R1336Q	This study
pCS27-SeHdCas9-Q1338R	pCS27-SeHdCas9 harboring Q1338R	This study
pCS27-SeHdCas9-R1336Q/Q1338A	pCS27-SeHdCas9 harboring R1336Q and Q1338A	This study
pCS27-SeHdCas9-R1336A/Q1338R	pCS27-SeHdCas9 harboring R1336A and Q1338R	This study
pCS27-SeHdCas9-R1152A	pCS27-SeHdCas9 harboring R1152A	This study
pCS27-SeHdCas9-N1228A	pCS27-SeHdCas9 harboring N1228A	This study
pCS27-SeHdCas9-Q1229A	pCS27-SeHdCas9 harboring Q1229A	This study
pCS27-SeHdCas9-E1230A	pCS27-SeHdCas9 harboring E1230A	This study
pCS27-SeHdCas9-K1340A	pCS27-SeHdCas9 harboring K1340A	This study
pCS27-SeHdCas9-R1152L	pCS27-SeHdCas9 harboring R1152L	This study
pCS27-SeHdCas9-R1152N	pCS27-SeHdCas9 harboring R1152N	This study
pCS27-SeHdCas9-R1152Q	pCS27-SeHdCas9 harboring R1152Q	This study
pCS27-SeHdCas9-R1152E	pCS27-SeHdCas9 harboring R1152E	This study
pCS27-SeHdCas9-R1152F	pCS27-SeHdCas9 harboring R1152F	This study
pCS27-SeHdCas9-Q1229R	pCS27-SeHdCas9 harboring Q1229R	This study
pCS27-SeHdCas9-Q1229E	pCS27-SeHdCas9 harboring Q1229E	This study
pCS27-SeHdCas9-Q1229F	pCS27-SeHdCas9 harboring Q1229F	This study
pCS27-SeHdCas9-Q1229I	pCS27-SeHdCas9 harboring Q1229I	This study

pCS27-SeHdCas9-W1337A	pCS27-SeHdCas9 harboring W1337A	This study
pCS27-SeHdCas9-W1337V	pCS27-SeHdCas9 harboring W1337V	This study
pCS27-SeHdCas9-W1337Y	pCS27-SeHdCas9 harboring W1337Y	This study
pCS27-SeHdCas9-S1227R	pCS27-SeHdCas9 harboring S1227R	This study
pCS27-SeHdCas9-K1322R	pCS27-SeHdCas9 harboring K1322R	This study
pCS27-SeHdCas9-I1334R	pCS27-SeHdCas9 harboring I1334R	This study
pCS27-SeHdCas9-K1340R	pCS27-SeHdCas9 harboring K1340R	This study
pCS27-SeHdCas9-W1337A/Q1229R	pCS27-SeHdCas9 harboring W1337A and Q1229R	This study
pCS27-SeHdCas9-Q1229R/K1322R	pCS27-SeHdCas9 harboring Q1229R and K1322R	This study
pCS27-SeHdCas9-Q1229R/K1340R	pCS27-SeHdCas9 harboring Q1229R and K1340R	This study
pCS27-SeHdCas9-Q1229R/R1336A	pCS27-SeHdCas9 harboring Q1229R and R1336A	This study
pCS27-SeHdCas9-Q1229R/E1230A	pCS27-SeHdCas9 harboring Q1229R and E1230A	This study
pCS27-SeHdCas9-Q1229R/E1230Y	pCS27-SeHdCas9 harboring Q1229R and E1230Y	This study
pCS27-SeHdCas9-Q1229R/E1230F	pCS27-SeHdCas9 harboring Q1229R and E1230F	This study
pCS27-SeHdCas9-K1322R/W1337A	pCS27-SeHdCas9 harboring K1322R and W1337A	This study
pCS27-SeHdCas9-K1322R/K1340R	pCS27-SeHdCas9 harboring K1322R and K1340R	This study
pCS27-SpdRY	pCS27 containing *Plpp1* promoter controlled SpdCas9 variant SpdRY with D10A/H840A/A61R/L1111R/D1135L/S1136W/G1218K/E1219Q/N1317R/A1322R/R1333P/R1335Q/T1337R	[4, 5]

pCS27-SpdNG-LWQT	pCS27 containing *Plpp1* promoter controlled SpdCas9 variant SpdNG-LWQT with D10A/H840A/V1135L/S1136W/R1333Q/V1335T	[4]
pETDuet-SeHCas9-R1336A	pETDuet-1 containing SeHCas9 with R1336A	This study
pETDuet-SeHCas9-R1152A	pETDuet-1 containing SeHCas9 with R1152A	This study
pETDuet-SeHCas9-Q1229A	pETDuet-1 containing SeHCas9 with Q1229A	This study
pETDuet-SeHCas9-Q1229R	pETDuet-1 containing SeHCas9 with Q1229R	This study
pETDuet-SeHCas9-K1322R/K1340R	pETDuet-1 containing SeHCas9 with K1322R and K1340R	This study
pZE-NNN-eGFP-sgRNA	pZE-eGFP-sgRNA containing NNN trinucleotides inserted after the start codon of eGFP	[4]
pZE-eGFP-sgTAA	Mutated the sgRNA in pZE-eGFP-sgRNA to target the TAA in eGFP sequence closest to the start codon as PAM	[4]
pZE-eGFP-sgTAG	Mutated the sgRNA in pZE-eGFP-sgRNA to target the TAG in eGFP sequence closest to the start codon as PAM	[4]
pZE-eGFP-sgTGA	Mutated the sgRNA in pZE-eGFP-sgRNA to target the TGA in eGFP sequence closest to the start codon as PAM	[4]
pZE-AAGN-eGFP-sgRNA	pZE-eGFP-sgRNA containing ATNCTT inserted after the start codon of eGFP	[4]

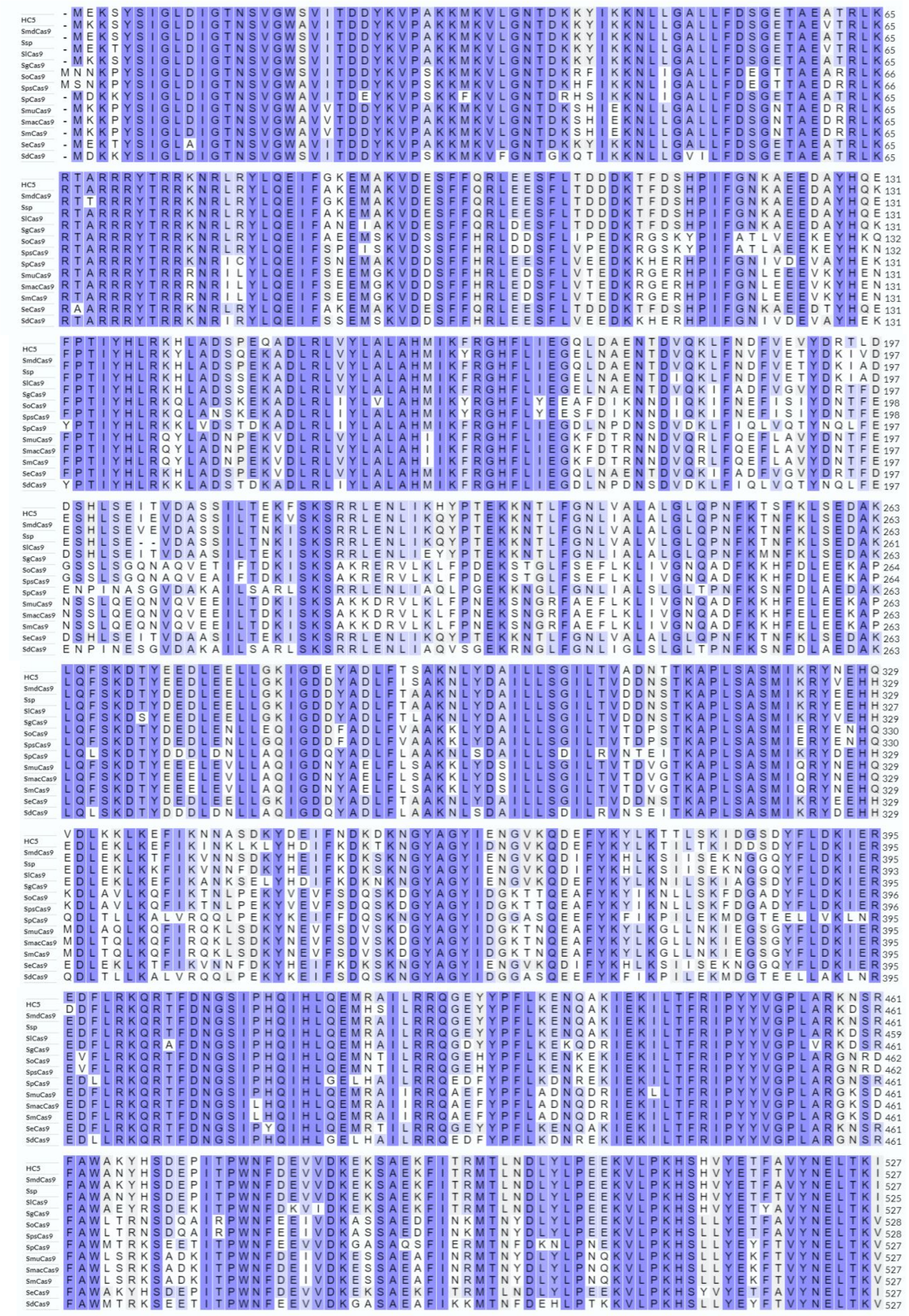
HC5
SmdCas9
Ssp
SlCas9
SgCas9
SoCas9
SpsCas9
SpCas9
SmuCas9
SmacCas9
SmCas9
SeCas9
SdCas9

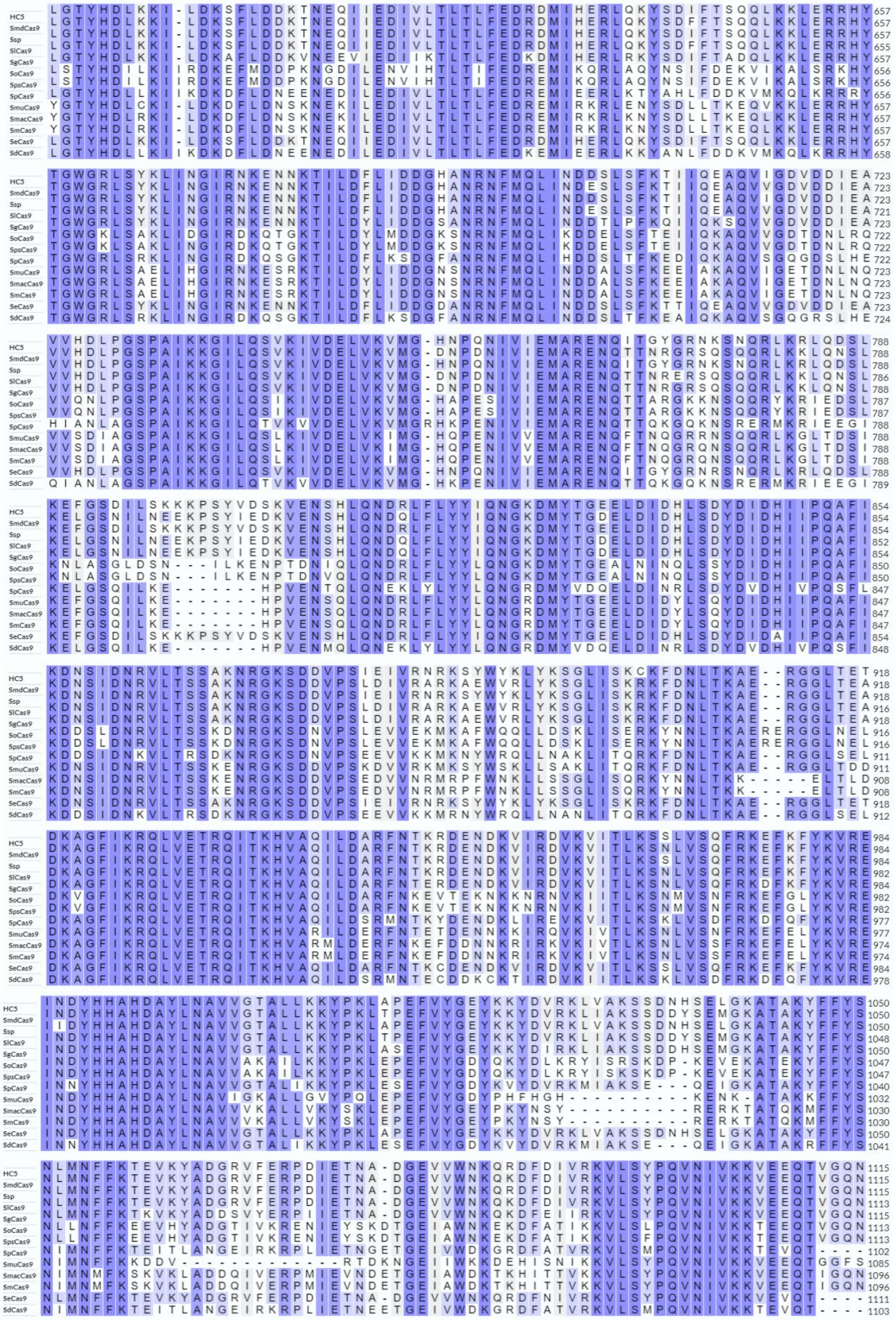

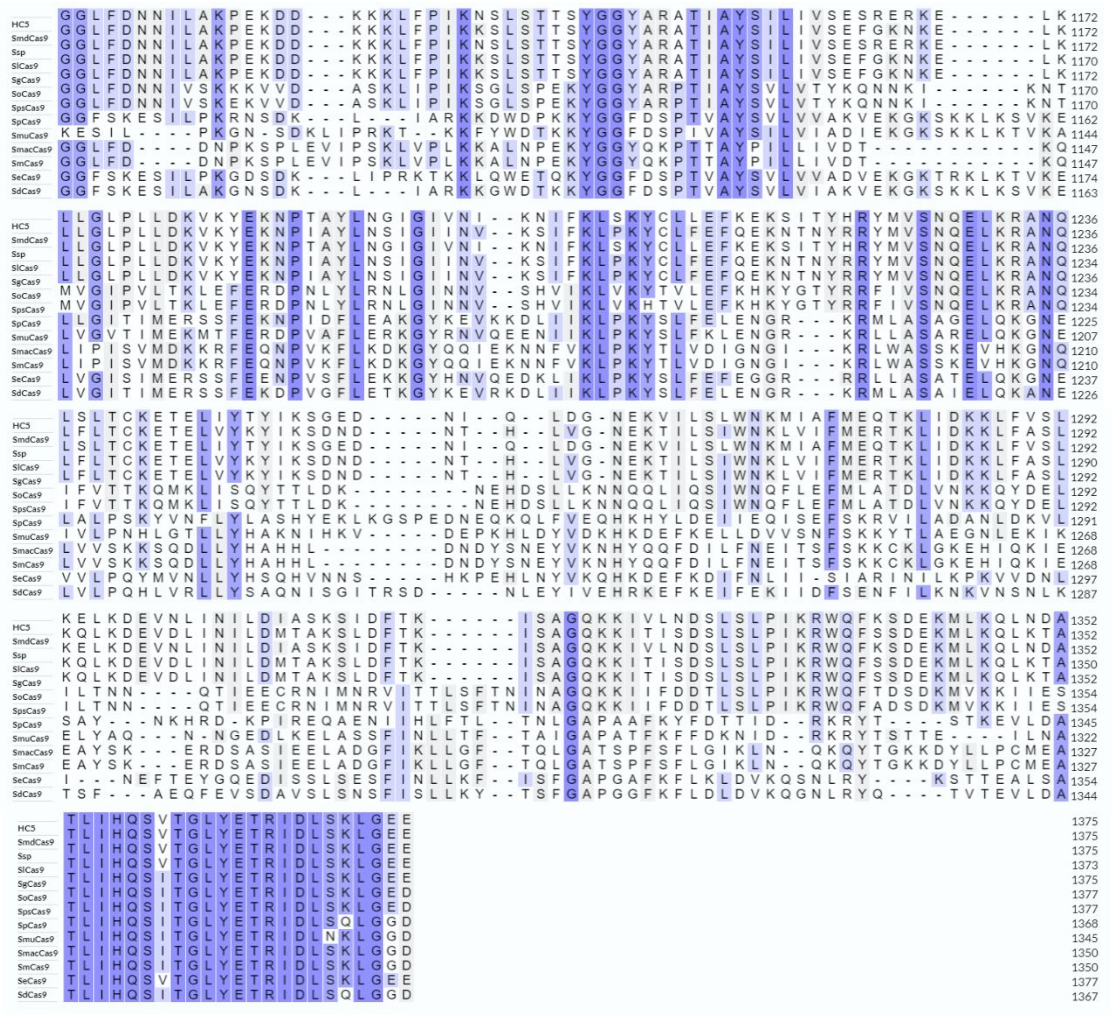

Figure S1. Sequence alignment of SpCas9-like orthologs with different PI motifs.

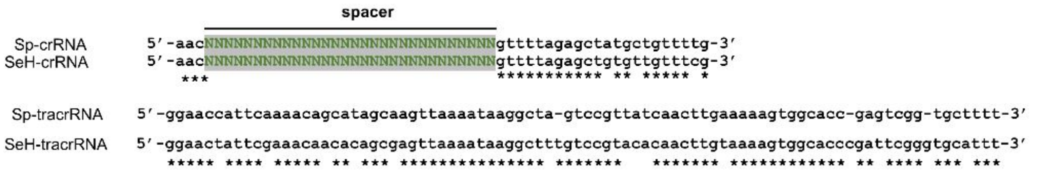

Figure S2. Sequence similarity of crRNA and tracrRNA between Sp-sgRNA and SeH-sgRNA

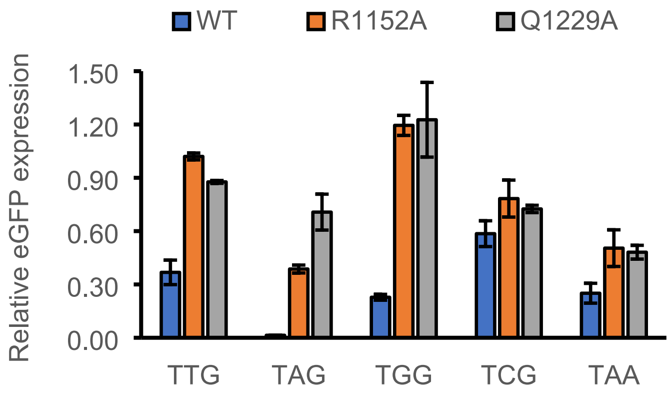

Figure S3. SeHdCas9-R1152A and -Q1229A on NNG and NAA PAMs

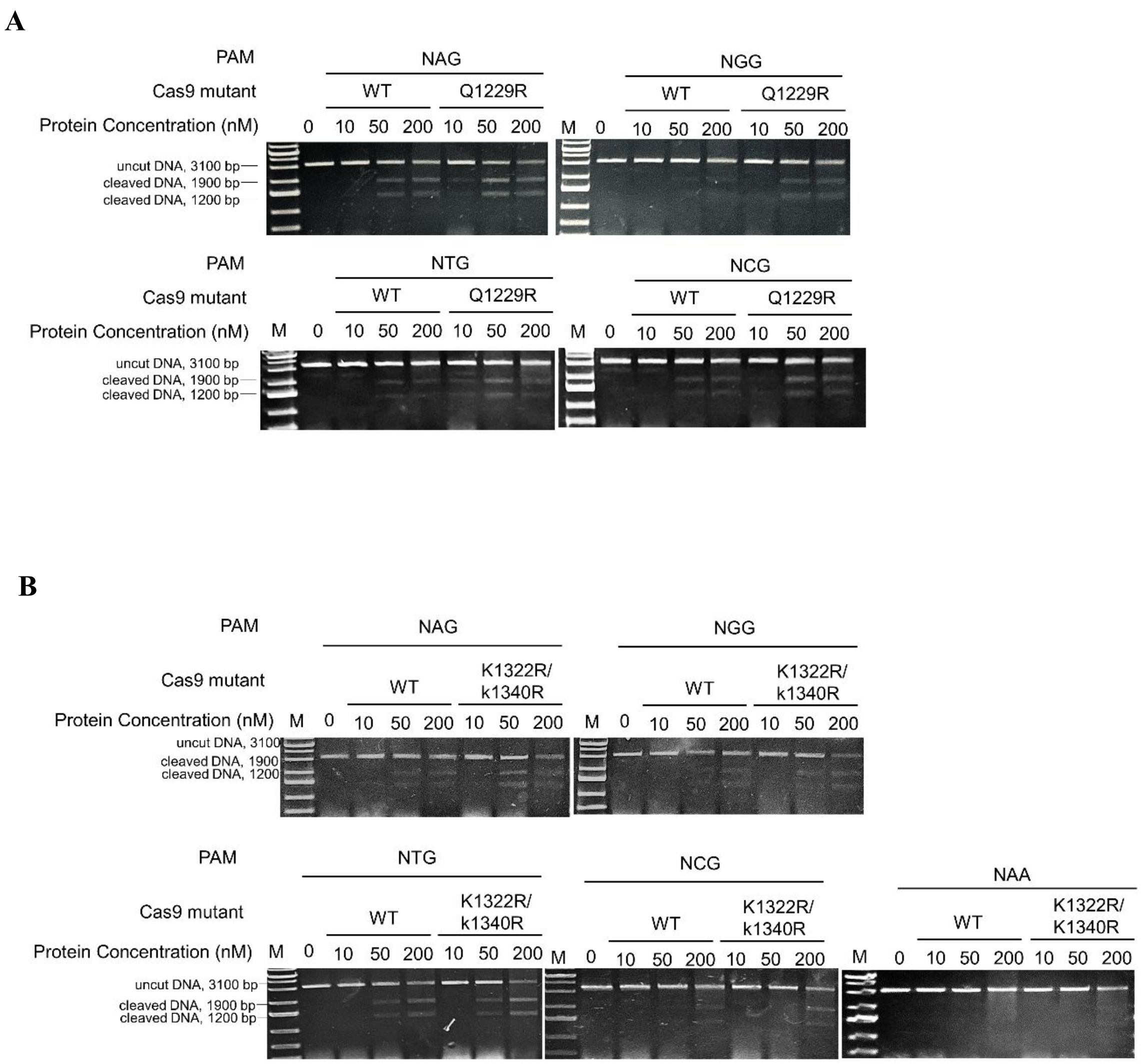

Figure S4. ***In vitro*** **DNA cleavage of SeHCas9-Q1229R and SeHCas9-RR against NNG and NAA PAMs**

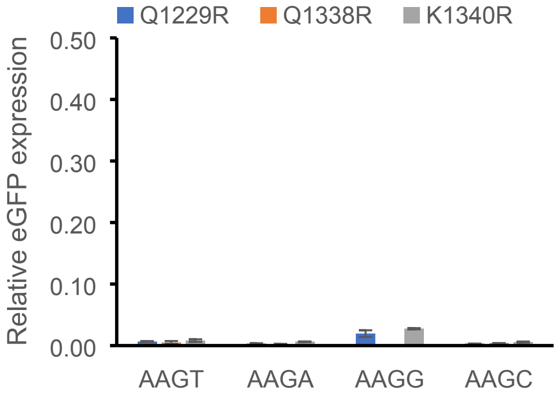

Figure S5. SeHdCas9 variants on AAGN PAMs

Reference

1. Atsumi, S.; Cann, A. F.; Connor, M. R.; Shen, C. R.; Smith, K. M.; Brynildsen, M. P.; Chou, K. J.; Hanai, T.; Liao, J. C., Metabolic engineering of Escherichia coli for 1-butanol production. *Metabolic engineering* **2008,** *10* (6), 305-311.
2. Shen, C. R.; Liao, J. C., Metabolic engineering of Escherichia coli for 1-butanol and 1-propanol production via the keto-acid pathways. *Metabolic engineering* **2008,** *10* (6), 312-320.
3. Lutz, R.; Bujard, H., Independent and tight regulation of transcriptional units in Escherichia coli via the LacR/O, the TetR/O and AraC/I1-I2 regulatory elements. *Nucleic acids research* **1997,** *25* (6), 1203-1210.
4. Wang, J.; Teng, Y.; Gong, X.; Zhang, J.; Wu, Y.; Lou, L.; Li, M.; Xie, Z.-R.; Yan, Y., Exploring and engineering PAM-diverse Streptococci Cas9 for PAM-directed bifunctional and titratable gene control in bacteria. *Metabolic Engineering* **2023,** *75*, 68-77.
5. Wang, J.; Teng, Y.; Zhang, R.; Wu, Y.; Lou, L.; Zou, Y.; Li, M.; Xie, Z. R.; Yan, Y., Engineering a PAM-flexible SpdCas9 variant as a universal gene repressor. *Nature Communications* **2021,** *12* (1), 68-77.
